# Synergistic probiotic consortium of *Bacillus subtilis* and *Lactobacillus fermentum* enhances palm kernel meal utilization and functional feed potential in poultry

**DOI:** 10.14202/vetworld.2025.3447-3463

**Published:** 2025-11-23

**Authors:** Mirnawati Mirnawati, Sindu Akhadiarto, Harnentis Harnentis, Gita Ciptaan, Zurmiati Zurmiati, Gusri Yanti, Anifah Srifani

**Affiliations:** 1 Department of Animal Nutrition and Feed Technology, Faculty of Animal Science, Universitas Andalas, Padang 25163, Indonesia; 2 Research Center for Animal Husbandry, National Research and Innovation Agency (BRIN), Bogor 16915, Indonesia; 3 Department of Agricultural Extension, Faculty of Social, Science and Education, Prima Nusantara Bukittinggi University, Bukittinggi 26122, Indonesia; 4 Doctoral Student of Animal Nutrition and Feed Technology, Faculty of Animal Science, Universitas Andalas, Limau Manis, Padang 25163, Indonesia

**Keywords:** *Bacillus subtilis*, consortium, enzyme activity, *Lactobacillus fermentum*, palm kernel meal, poultry feed, probiotic

## Abstract

**Background and Aim:**

Palm kernel meal (PKM), a major by-product of the palm oil industry, is rich in nutrients but poorly utilized in poultry feed due to its high fiber and mannan content. Improving PKM digestibility through microbial bioconversion could reduce dependency on expensive protein sources, such as soybean meal. This study aimed to evaluate a consortium of *Bacillus subtilis* and *Lactobacillus fermentum* for its enzymatic activity, probiotic properties, and potential to enhance PKM utilization in poultry diets.

**Materials and Methods:**

The research was performed in four stages: (1) measurement of cellulase, mannanase, and protease activities in individual and combined bacterial cultures (seven treatments, five replications); (2) determination of enzyme activities in *B. subtilis* and *L. fermentum* (1:1) grown in de Man, Rogosa, and Sharpe broth supplemented with 0%–20% PKM (four treatments, seven replications); (3) *in vitro* probiotic characterization, including acid and bile tolerance, hydrophobicity, autoaggregation, coaggregation, and pathogen inhibition; and (4) evaluation of enzyme activity in natural media composed of coconut water and shrimp wastewater. Data were analyzed using analysis of variance and Duncan’s multiple range tests at p < 0.05.

**Results:**

The 1:1 consortium exhibited the highest enzyme activities; cellulase (13.71 U/mL), mannanase (17.05 U/mL), and protease (9.32 U/mL). The consortium retained high activity in 15% PKM media and demonstrated strong acid tolerance (70.6% survival at pH 2.5), bile salt tolerance (62.84% at 0.3%), and thermal resistance (83.15% at 42°C). It showed 83.75% hydrophobicity, 73.32%–71.64% autoaggregation, and 78.13% coaggregation, along with marked inhibition against *Escherichia coli*, *Salmonella* Enteritidis, and *Staphylococcus aureus* (15.07–17.12 mm inhibition zones). Natural media composed of 70% coconut water + 30% shrimp wastewater supported optimal enzymatic performance.

**Conclusion:**

The *B. subtilis*–*L. fermentum* consortium demonstrates potent synergistic enzymatic and probiotic traits, indicating its suitability as a bioenhancer for PKM-based poultry feed. This dual-function probiotic could lower feed costs, improve nutrient digestibility, and support sustainable poultry production. Future work should validate these results through *in vivo* trials and large-scale fermentation optimization.

## INTRODUCTION

Indonesia is among the world’s largest producers of palm oil, with approximately 15,081,021 ha of oil palm plantations recorded in 2021. During the processing of oil palm fruit bunches, a by-product known as palm kernel meal (PKM) is generated, constituting about 3.5% of each fresh fruit bunch or around 45%–46% of total palm oil production. Consequently, PKM output is estimated to reach approximately 22.37 million tons annually, representing a valuable yet underutilized feed resource for poultry.

Nutritionally, PKM contains 0.27% calcium, 0.94% phosphorus, 48.4 ppm copper, 17.31% crude protein, 27.62% crude fiber, and 7.14% crude fat [1]. Although the crude protein level is relatively high, PKM is still not widely used in poultry diets. PKM can replace up to 40% of the soybean meal in broiler feeds [2]. However, prior processing is essential to improve its quality because PKM has limited digestibility due to its high hemicellulose fraction, approximately 57.8% of which consists of β-mannan [3]. Poultry lack endogenous mannan-degrading and cellulolytic enzymes, leading to inefficient nutrient utilization. One approach to overcoming this limitation involves supplementing with probiotics that produce cellulase and mannanase enzymes, which can reduce the crude fiber and mannan content, thereby enhancing PKM’s nutritional quality.

Among cellulolytic and mannanolytic microbes, *Bacillus subtilis* has shown great potential. Mirnawati *et al*. [4] reported that PKM fermented with *B. subtilis* for 6 days increased crude protein content by 24.65%, crude fiber digestibility by 53.25%, and nitrogen retention by 68.47%. The bacterium produced mannanase, cellulase, and protease activities of 24.27, 17.13, and 10.27 U/mL, respectively [5], and could be incorporated into broiler diets up to 25% without negative effects [6]. Similarly, *Lactobacillus fermentum* isolated from decomposed PKM demonstrated cellulase, mannanase, and protease activities of 18.84, 24.86, and 10.45 U/mL, respectively. Fermentation of PKM with *L. fermentum* improved crude protein content by 25.81%, reduced crude fiber by 16.90%, and increased metabolic energy to 2743 kcal/kg [7].

Most previous studies on PKM fermentation or probiotic application have utilized single bacterial strains, such as *B. subtilis* or *L. fermentum*. In contrast, the present study investigates a defined consortium of these two bacteria at varying ratios to explore possible synergistic effects on enzyme production and probiotic performance. Both strains have demonstrated strong potential in reducing cellulose and mannan content in feed, but their combination as a probiotic consortium could provide broader functional benefits. In this study, *B. subtilis* and *L. fermentum* were employed as cellulase- and phytase-producing probiotics capable of improving PKM digestibility while conferring health-promoting effects. The administration of probiotics aims to stabilize the gastrointestinal microbial community in monogastric animals and to enhance digestive efficiency through the production of beneficial enzymes [8, 9]. In addition, probiotics are increasingly used as safe alternatives to antibiotics, whose prolonged use can pose significant risks to human health.

The use of bacterial consortia as probiotics, rather than single-strain formulations, is based on the principle that multi-strain or multi-species combinations can deliver broader, more synergistic effects on poultry health and feed utilization. A *B. subtilis*–*L. fermentum* consortium combines complementary metabolic activities, including enhanced enzyme secretion (e.g., mannanase and cellulase), improved pathogen inhibition, and more effective modulation of the intestinal microbiota compared with individual strains [10]. Members of such a consortium can target diverse substrates, inhibit various pathogens, and support each other’s growth and activity within the gastrointestinal tract through metabolic complementarity. This synergism fosters a more balanced and resilient gut ecosystem, resulting in improved nutrient absorption, growth performance, immune response, and overall poultry productivity [11].

Despite Indonesia’s abundant production of PKM as a palm oil by-product, its direct utilization in poultry feed remains limited due to high levels of indigestible β-mannan and crude fiber. Numerous studies have demonstrated the benefits of single microbial fermentations using either *B. subtilis* or *L. fermentum* in improving the nutritional quality of PKM. However, the majority of these investigations have focused on single-strain fermentations and have not examined the synergistic potential of combining cellulolytic and mannanolytic bacteria. The enzymatic interactions, co-metabolic mechanisms, and probiotic resilience of multi-strain consortia remain poorly understood in the context of PKM bioconversion. Furthermore, while several studies have established the *in vitr*o enzyme activities of individual strains, there is limited evidence evaluating their combined probiotic characteristics, such as acid and bile tolerance, aggregation behavior, hydrophobicity, and pathogen inhibition. In addition, information on the use of sustainable, low-cost natural media for the cultivation of such probiotic consortia (e.g., coconut water and shrimp wastewater) is scarce. Therefore, a comprehensive evaluation of a *B. subtilis* and *L. fermentum* consortium in terms of enzymatic activity, probiotic functionality, and alternative growth media is essential to establish its biotechnological potential for improving PKM digestibility and poultry feed efficiency.

This study aimed to develop and characterize a probiotic consortium composed of *B. subtilis* and *L. fermentum* to enhance the utilization of PKM in poultry feed. Specifically, the objectives were to:

(1) Evaluate the enzymatic activity (cellulase, mannanase, and protease) of individual bacterial strains and their combinations at different ratios
(2) Determine the effect of PKM concentration on enzyme activity in culture media
(3) Assess the *in vitro* probiotic characteristics of the consortium, including acid and bile tolerance, hydrophobicity, autoaggregation, coaggregation, and antimicrobial activity against common poultry pathogens (*Escherichia coli*, *Salmonella* Enteritidis, and *S. aureus*); and
(4) Explore the potential of alternative natural media composed of coconut water and shrimp wastewater to support enzyme production and bacterial growth.

By integrating enzymatic and probiotic evaluations, the study seeks to establish a dual-functional, eco-friendly microbial consortium capable of improving PKM quality, reducing feed costs, and promoting sustainable poultry production through the replacement of conventional protein sources and antibiotic growth promoters.

## MATERIALS AND METHODS

### Ethical approval

This study did not involve live animals or human participants. All experimental procedures were conducted using bacterial isolates and *in vitro* analyses in accordance with institutional biosafety and bioethics guidelines of the Faculty of Animal Science, Universitas Andalas, Indonesia. All biosafety and waste disposal procedures adhered strictly to the recommendations of the Universitas Andalas Ethics Committee to ensure environmental and personnel safety.

### Study period and location

The study was conducted from December 2023 to June 2024 at the Field Laboratory, Faculty of Animal Science, Universitas Andalas, Indonesia.

### Source of isolates

Two bacterial isolates were used in this study: *L. fermentum*, previously isolated from PKM in June 2021, and *B. subtilis*, obtained from a commercially available culture (National Scientific Research Agency, Indonesia).

### Bacterial characterization

The isolates were characterized using a series of biochemical tests, including oxidase, catalase, and methyl red (MR)–Voges Proskauer (VP) reactions, to confirm their basic physiological and metabolic traits.

### Bacterial identification

Genomic DNA was extracted from previously harvested bacterial pellets using a Lysis Solution and Proteinase K, followed by incubation at 56°C for 30 min. RNAse A treatment was carried out at room temperature for 10 min, after which 50% cold ethanol was added, and the mixture was transferred to a spin column. DNA was washed sequentially with Wash Buffers I and II, eluted with 50 μL elution buffer, and stored at −20°C.

DNA quality and integrity were verified by electrophoresis on a 1% agarose gel containing ethidium bromide in Tris-Borate-EDTA buffer, using λ DNA as a marker, and visualized under an ultraviolet (UV) transilluminator (Biometra, Germany). DNA concentration was determined with a Biodrop UV–visible spectrophotometer (BioDrop Ltd., Cambridge, UK). Polymerase chain reaction (PCR) amplification of the 16S ribosomal RNA (*16S rRNA*) gene was performed using KOD One Blue Master Mix (Toyobo, Japan) and specific primers. PCR products were separated on a 1% agarose gel alongside a 1-kb DNA ladder, visualized under UV light, and the remaining PCR mixtures were stored at −20°C for subsequent analysis.

### Experimental workflow

**Figure 1:**
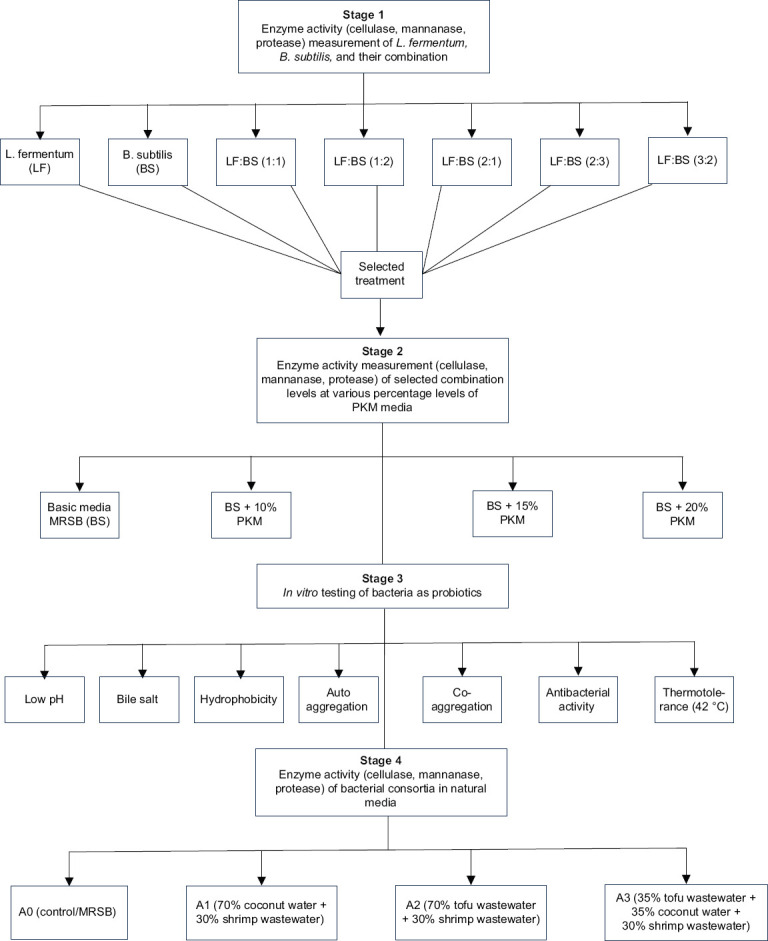
The workflow of the study.

This study consisted of four stages, summarized in the workflow diagram (Figure 1).

### Stage 1: Enzyme activity measurement of *L. fermentum*, *B. subtilis*, and their combination

The enzyme activities measured included cellulase, mannanase, and protease. Seven treatments with five replications each were conducted using *L. fermentum*, *B. subtilis*, and their combinations at ratios of 1:1, 1:2, 2:1, 2:3, and 3:2.

#### Cellulase activity

Cellulase activity was determined following the method of Jennifer and Thiruneelakandan [12]. A mixture of 1 mL enzyme supernatant and 1 mL of extract (0.5 g carboxymethyl cellulose+ 10 mL acetate buffer) was incubated for 30 min at 40°C, pH 5, in a water bath shaker (90 rpm). One mL of Nelson AB reagent was then added to 1 mL of boiling water and incubated for 20 min. After cooling, 1 mL of phosphomolybdate and 7 mL of distilled water were added, and the absorbance was measured at 575 nm.

One unit (U) of cellulase activity was defined as the amount of enzyme releasing 1 μmol glucose equivalents min^−1^ under assay conditions.


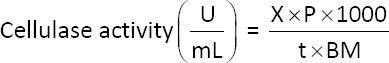


Where X = Standard curve conversion result, P = Dilution, t = Incubation time (min), and BM = Molecular weight of glucose.

#### Mannanase activity

Mannanase activity was determined using a modified method by El-Naggar *et al*. [13]. The reaction mixture contained 0.5 mL of 50 mM potassium phosphate buffer (pH 7.0), 1% locust bean gum, and 0.5 mL of enzyme supernatant. The mixture was incubated at 45°C for 60 min. The amount of reducing sugar released was quantified using the dinitrosalicylic acid (DNS) method. One unit of mannanase activity corresponded to the release of 1 μmol mannose/min under assay conditions.


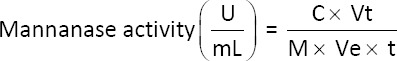


Where C = Reducing sugar concentration (μmol/mL) from the DNS standard curve, Vt = Total reaction volume (mL), M = Molecular weight of mannose, Ve = Enzyme volume (mL), and t = Incubation time (min).

#### Protease activity

Protease activity was measured using the modified method of Prihatiningsih *et al*. [14]. A mixture of 0.5 mL of 0.6% (w/v) casein substrate in 0.1 M Tris-HCl buffer (pH 8.0) and 0.1 mL of enzyme solution was incubated at 45°C for 30 min with shaking (90 rpm). The reaction was stopped by adding 0.5 mL of cold trichloroacetic acid and centrifuging at 1,800 × *g* for 15 min at 4°C. The absorbance of the supernatant was measured at 275 nm using a spectrophotometer.

Tyrosine (1–120 μg/mL) was used to generate a standard curve, and one unit of protease activity (U) was defined as the amount of enzyme releasing 1 μg tyrosine/min/mL of enzyme solution.


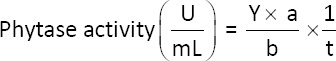


Where Y = Sample absorbance, a = Intercept, b = Slope of regression curve (Y = a + bx), and t = Incubation time (min).

### Stage 2: Enzyme activity of selected combinations in different PKM concentrations

To determine the optimal growth conditions for the *B. subtilis*–*L. fermentum* consortium, de Man, Rogosa, and Sharpe broth (MRSB) was used as the basal medium supplemented with 0%, 10%, 15%, and 20% PKM. Four treatments with seven replications were applied, and cellulase, mannanase, and protease activities were evaluated.

### Stage 3: *In vitro* evaluation of bacterial combinations as probiotics

#### Low pH survival

The acid tolerance test followed the modified method of Dowarah *et al*. [15]. MRSB was adjusted to pH 2.5 with 37% HCl, while unacidified de Man, Rogosa, and Sharpe (MRS) (pH 6.8) served as the control. Each medium (5 mL) was inoculated with 0.5 mL of bacterial culture and incubated at 37°C for 3 and 6 h (90 rpm). Absorbance was measured at 600 nm, and survival percentage was calculated as:

Survival (%) = (A_t_/A_0_) × 100

Where A_0_ is optical density (OD) at 600 nm (or colony-forming unit [CFU]/mL) at pH 6.8 and A_t_ after exposure (pH 2.5).

#### Resistance to bile salts

Bile tolerance was tested based on Srifani *et al*. [16]. MRSB medium containing 0%, 0.3%, and 0.5% oxgall bile salts was inoculated with 0.5 mL of the bacterial consortium and incubated at 37°C for 5 h (90 rpm). Growth was measured at 600 nm in triplicate. The percentage survival was calculated using the formula of Tokatli *et al*. [17]:

Survival (%) = (A_t_/A_0_) × 100

Where A_0_ is OD at 600 nm (or CFU/mL) at 0% bile salt and A_t_ after exposure (0.3% and 0.5% bile salt).

#### Hydrophobicity assay

Hydrophobicity was determined following Fadda *et al*. [18]. MRSB medium (0.68 g in 100 mL distilled water) was autoclaved and inoculated with 1 mL of the bacterial consortium, then incubated for 24 h at 37°C with shaking (90 rpm). After incubation, bacterial adhesion to stainless steel was assessed by measuring the absorbance of the adhered (A) and non-adhered (Ao) cells at 600 nm.


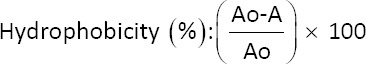


Where Ao = OD value in the liquid phase and A = OD value attached to stainless steel.

#### Autoaggregation assay

Autoaggregation was assessed using the method of Polak-Berecka *et al*. [19]. Bacterial suspensions (10^8^ CFU/mL) in phosphate-buffered saline (pH 7.2) were vortexed, and the initial absorbance (ODi) was measured at 600 nm. After 2 h incubation at 37°C, the final absorbance (OD_2_h) was recorded.

A_ct_ (%) = [1 – (OD_2h_ – ODi)] × 100

Where ODi = Initial optical density of the microbial suspension at 600 nm, OD2h = Optical density of the microbial suspension at 600 nm after 2 h.

#### Coaggregation assay

Coaggregation between *L. fermentum* and *B. subtilis* was performed following Reuben *et al*. [20]. Equal volumes (2 mL each) of both bacterial suspensions (10^8^ CFU/mL) were mixed and incubated for 2 h at 37°C. The absorbance of the mixture (ODmix) was measured at 600 nm and compared with the individual controls.

Co-aggregation = [1−ODmix/(OD *Lactobacillus fermentum* + OD *Bacillus subtilis*)/2] × 100

#### Antibacterial activity against pathogenic bacteria

Antagonistic activity against *E. coli*, *Salmonella* Enteritidis, and *S. aureus* was assessed using the paper disc diffusion method as modified from Pisol *et al*. [21]. Nutrient agar was inoculated with 0.2% pathogenic bacteria, and sterile paper discs soaked in bacterial supernatant were placed on the surface. Plates were incubated at 37°C for 24 h, and inhibition zones were measured with calipers following Winastri *et al*. [22].

#### Thermotolerance (42°C)

Thermal resistance was tested according to Kumar *et al*. [23]. The consortium was cultured on MRS agar and incubated for 24–48 h at both 37°C and 42°C. Bacterial growth was measured spectrophotometrically, and survival was calculated as:

Survival (%) = (A_t_/A_0_) × 100

Where A_0_ is OD600 (or CFU/mL) at 37°C and A_t_ after exposure (42°C).

### Stage 4: Enzyme activity of bacterial consortium in natural media

At this stage, the MRSB medium was replaced with various natural and low-cost media. Four treatments were applied:

- A0: Control (MRSB)
- A1: 70% coconut water + 30% shrimp wastewater
- A2: 70% tofu wastewater + 30% shrimp wastewater
- A3: 35% tofu wastewater + 35% coconut water + 30% shrimp wastewater.

Each treatment had seven replications, and cellulase, mannanase, and protease activities were determined.

### Statistical analysis

The study comprised four stages, each with different experimental designs.

- Stage 1: Completely randomized design (CRD) with seven treatments and five replications
- Stage 2: CRD with four treatments and seven replications
- Stage 3: Descriptive analysis for *in vitro* probiotic tests
- Stage 4: CRD with four treatments and seven replications.

Data were analyzed using IBM Statistical Package for the Social Sciences Statistics 26.0 (IBM Corp., NY, USA). Normality was verified by the Shapiro–Wilk test, and homogeneity of variances by Levene’s test before performing one-way analysis of variance. Mean differences were determined using Duncan’s multiple range test, with statistical significance set at p < 0.05, following the procedure of Steel and Torrie [24].

## RESULTS

### Characterization of the Y1 isolate (*L. fermentum*)

The characterization of the Y1 isolate (*L. fermentum*) is presented in Table 1. table summarizes the key biochemical and morphological characteristics observed during the identification process.

**Table 1:** Bacterial characterization of Y1 (*Lactobacillus fermentum*).

Characterization	Y1 isolate
Gram	+
Aerobic/Anaerobic	Aerobic
Triple sugar iron agar	M
Gas	−
H_2_S	−
Catalase	−
Oxidase	−
Mortality	−
Indole	−
Urea	−
Citrate	−
Lactose	−
Glucose	−
Sucrose	−
Mannitol	−
Methyl red	+
Voges Proskauer	+
Oxidative-fermentative	−
Arginine	−
Maltose	−
Melezitose	−
Melibiose	−
Arabinose	−
Raffinose	−
Sorbitol	−
Trehalase	−
Genus	*Lactobacillus* spp.

### Molecular identification of the Y1 isolate

**Figure 2:**
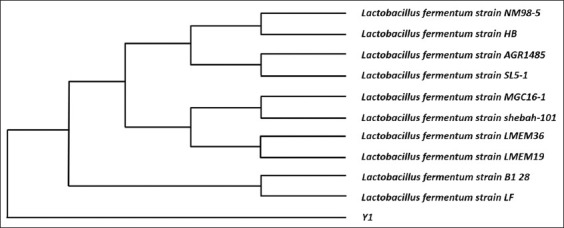
The phylogenetic tree of Y1 isolate based on 16S ribosomal RNA gene sequences.

Phylogenetic analysis of the Y1 isolate was performed using the neighbor-joining method with a bootstrap value of 1000 to ensure the reliability of branching patterns. Evolutionary distances were calculated using the Kimura two-parameter model. Based on the *16S rRNA* gene sequence, the resulting phylogenetic tree (Figure 2) demonstrates the evolutionary relationship between the Y1 isolate and ten closely related reference bacterial species obtained from the GenBank database.

### Enzyme activity of *L. fermentum*, *B. subtilis*, and their combination

The enzyme activity results for *B. subtilis* and *L. fermentum*, both individually and in combination, at different treatment levels are presented in Table 2. Among the tested treatments, the A1B1 combination produced the highest cellulase and mannanase enzyme activities, while treatment A exhibited the highest protease activity.

**Table 2:** Cellulase, mannanase, and protease activities of *L. fermentum*, *B. subtilis*, and their combinations.

Enzyme activity	Treatments						

	A	B	A1B1	A1B2	A2B1	A2B3	A3B2
Cellulase	12.48 ± 0.162^bc^	6.21 ± 0.340^a^	13.71 ± 0.129^d^	12.57 ± 0.167^bc^	11.91 ± 0.245^b^	12.92 ± 0.239^c^	11.88 ± 0.230^b^
Mannanase	12.43 ± 0.324^b^	6.79 ± 0.121^a^	17.05 ± 0.221^d^	13.72 ± 0.401^c^	14.29 ± 0.242^c^	14.03 ± 0.168^c^	12.24 ± 0.119^b^
Protease	11.29 ± 0.049^e^	9.2 ± 0.119^d^	9.32 ± 0.053^d^	7.26 ± 0.045^c^	7.33 ± 0.228^c^	6.48 ± 0.396^b^	5.64 ± 0.030^a^

Note: Values are means and standard errors of five replicates. Mean values in the same row with different letters are significantly different (p < 0.05). Treatments were as follows: A (*L. fermentum*), B (*B. subtilis*), A1B1 (*B. subtilis*: *L. fermentum* with a comparison level of 1:1), A1B2 (*B. subtilis*: *L. fermentum* with a comparison level of 1:2), A2B1 (*B. subtilis*: *L. fermentum* with a comparison level of 2:1), A2B3 (*B. subtilis*: *L. fermentum* with a comparison level of 2:3), and A3B2 (*B. subtilis*: *L. fermentum* with a comparison level of 3:2). *B. subtilis* = B*acillus subtilis*, *L. fermentum* = L*actobacillus fermentum.*

### Enzyme activity of selected combination (1:1) at different PKM concentrations

**Figure 3:**
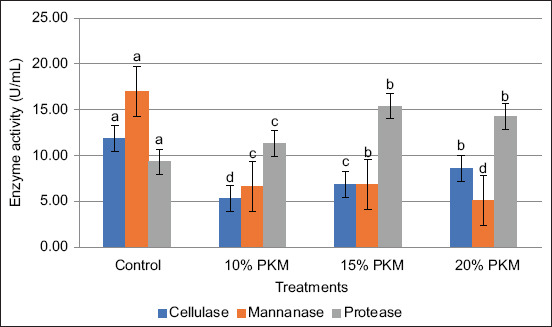
Enzyme activity of *Lactobacillus fermentum* and *Bacillus subtilis* (1:1) in de Man, Rogosa, and Sharpe broth media + various levels of Palm kernel meal.

The enzyme activities of the *L. fermentum* and *B. subtilis* consortium (1:1) cultured in MRSB medium supplemented with varying levels of PKM are shown in Figure 3. The treatment containing 20% PKM yielded the highest cellulase activity, whereas the 15% PKM treatment demonstrated the greatest mannanase and protease enzyme activities.

### *In vitro* evaluation of bacterial consortium as a probiotic candidate

The results of *in vitro* probiotic testing of the bacterial consortium are summarized in Table 3. The bacterial consortium exhibited a survival rate of 62.48% at 0.3% bile salt concentration, which decreased to 46.23% at 0.5% bile salt. The hydrophobicity value of the consortium was recorded at 83.74%. Moreover, the bacterial consortium maintained a survival rate of 68.10% at pH 2.5 after 6 h of incubation. Antagonistic activity tests revealed inhibition zones of 15.07 mm against *E. coli*, 14.12 mm against *Salmonella* Enteritidis, and 17.12 mm against *S. aureus*, indicating notable antipathogenic potential.

**Table 3:** *In vitro* testing of probiotic candidates.

*In vitro* testing of probiotics	Value
Hemolytic test	-
Resistance to pH 2.5 (%)	
3 h	70.60 ± 0.56
6 h	68.10 ± 0.18
Resistance to bile salts	
0.3%	62.84 ± 1.60
0.5%	46.23 ± 1.17
Hydrophobicity (%)	83.75 ± 0.27
Autoaggregation of *Lactobacillus fermentum*	73.32 ± 0.74
Autoaggregation of *Bacillus subtilis*	71.64 ± 0.83
Co-aggregation	78.13 ± 0.48
Resistance to pathogenic bacteria (mm)	
*Escherichia coli*	15.07 ± 0.70
*Salmonella* Enteritidis	14.12 ± 1.10
*Staphylococcus aureus*	17.12 ± 1.06
Thermoresistance (42°C)	83.15 ± 1.78

Note: Values are means and standard errors. Means in the same column with different letters are significantly different (p < 0.05).

### Enzyme activity of bacterial consortium in natural media

**Figure 4:**
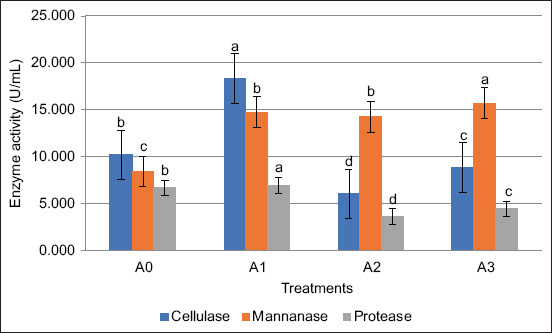
Enzyme activity of *Lactobacillus fermentum*: *Bacillus subtilis* (1:1) in various natural media.

The enzyme activity profiles of the bacterial consortium cultured in different natural media are presented in Figure 4. The A1 treatment (70% coconut water + 30% shrimp wastewater) produced the highest cellulase and protease activities, while the A2 treatment (70% tofu wastewater + 30% shrimp wastewater) yielded the highest mannanase activity.

## DISCUSSION

### Characterization of the Y1 isolate

The Y1 isolate (*L. fermentum*) was identified as a Gram-positive bacterium and showed aerobic growth (Table 1). Gram-positive bacteria possess a thick peptidoglycan layer in their cell wall, typically tens of nanometers thick, which provides mechanical strength and is essential for cell viability, acting as a constraint to internal turgor pressure [25]. The isolate produced a red slant without gas or hydrogen sulfide on triple sugar iron agar (TSIA), indicating an alkaline reaction and lack of sulfur reduction, which is typical for *Lactobacillus* species that do not ferment sugars with gas production.

Biochemical testing revealed that Y1 was negative for catalase and oxidase. The isolate was also non-motile and did not produce indole, urea, or use citrate, further supporting its identification as a *Lactobacillus* species. In carbohydrate fermentation tests, Y1 was negative for lactose, glucose, sucrose, mannitol, maltose, melezitose, melibiose, arabinose, raffinose, sorbitol, and trehalose. However, it tested positive in the MR and VP tests, suggesting a mixed acid fermentation pathway. The oxidative-fermentative test was negative, and arginine was not utilized, which is in line with homofermentative lactic acid bacteria (LAB) that primarily produce lactic acid from hexose sugars without extensive secondary metabolism [26].

### Identification of the Y1 isolate

The Y1 isolate was confirmed as *L. fermentum* based on molecular identification (Figure 2). This species belongs to the LAB group, which is commonly recognized for its probiotic potential. Paek *et al*. [27] have reported that *L. fermentum* demonstrates strong tolerance to acidic and bile conditions as well as good adhesion ability to intestinal epithelial cells, which are important for survival and colonization in the gastrointestinal tract.

In addition, *L. fermentum* strains exhibit antagonistic effects against various pathogenic bacteria through the production of organic acids, hydrogen peroxide, and bacteriocins. Moreover, several strains have been associated with beneficial functions, such as immune response modulation and gut health improvement. Therefore, the confirmation of Y1 as *L. fermentum* highlights its potential as a probiotic candidate with functional applications in animal nutrition and health.

### Enzyme activity of *L. fermentum*, *B. subtilis*, and their combination

Some bacteria produce compounds or substances, namely enzymes, needed to aid the digestion process of certain food substrates in the digestive tract. Lactic acid-producing microbes can produce enzymes to aid digestion. Some enzymes that can be produced by bacteria are cellulase, mannanase, and protease enzymes.

Cellulase enzymes can break down crude fiber components, which are difficult to digest in the digestive tract of poultry. Microbe-derived mannanase enzymes are primarily capable of breaking down different mannan polymers to produce mannobiose and mannotriose without mannose [28, 29], and protease plays an important role in protein digestion [30]. In this study, *L. fermentum* and *B. subtilis* bacteria can produce cellulase, mannanase, and protease enzymes. The combination of these two bacteria was tested to determine their synergy.

Based on research data in Table 2, it is known that the treatment with *L. fermentum* bacteria alone produced the highest protease activity (11.29 U/mL), but combining *L. fermentum* + *B. subtilis* bacteria in a 1:1 ratio produced the highest cellulase activity (13.71 U/mL) and mannanase activity (17.05 U/mL). The *L. fermentum* and *B. subtilis* consortium synergistically enhances cellulase and mannanase activities compared with their solo cultures through several intertwined mechanisms. Both bacteria naturally produce these enzymes, but when combined, they complement each other by producing diverse enzyme isoforms that target different sites in the lignocellulosic substrate, leading to more efficient hydrolysis. Moreover, *L. fermentum* produces organic acids, such as lactic acid, that lower pH and create favorable conditions for enzyme activity and stability, which benefits the enzymes secreted by *B. subtilis*.

The interaction between the strains improves substrate degradation through metabolic cooperation, where one strain releases intermediate products that serve as substrates or inducers for the other, allowing continuous and enhanced enzyme secretion. This cross-feeding mechanism also reduces feedback inhibition, which can limit enzyme production in single strains. In addition, co-culturing increases total microbial biomass and enzyme secretion, as the mutualistic growth environment supports higher proliferation and metabolic activity. This multifaceted synergy ultimately results in significantly higher cellulase and mannanase activities in the consortium compared with single-strain cultures, highlighting the power of microbial cooperation in enzymatic biomass degradation.

Chukwuma *et al*. [31] reported that a consortium of Bacillus bacteria showed a cellulase activity of 0.78 U/mL at 38°C. Similarly, Kitaevskaya *et al*. [32] demonstrated strong proteolytic activity with LAB consortia (*Lactobacillus* *casei*, *L. fermentum*, *Lactobacillus* *plantarum*) with protein hydrolysis rates of 83.4% and 79.2% in different ratios, comparable to the protease activity observed in this study’s single-strain treatment. The mannanase activity reported here for the *L. fermentum* + *B. subtilis* consortium (17.05 U/mL) notably exceeds that reported by Christianah and Ajoke [33], who reported a maximum of 6.37 U/mL for *B. subtilis* alone after 72 h. These comparisons suggest that this study’s 1:1 consortium not only achieves a balance of enzyme production across cellulase, mannanase, and protease but also offers enhanced mannanase activity relative to previous reports. Therefore, the consortium treatment was selected for further study owing to its superior overall enzymatic profile and promising synergy between strains.

### Enzyme activity of selected combinations of PKM media at various percentage levels

The combination of *L. fermentum* + *B. subtilis* in a ratio of 1:1 was tested on MRSB media containing 0% (control), 10%, 15%, and 20% PKM substrates. This was done to determine the ability of the bacterial consortium to produce cellulase, mannanase, and protease enzymes in media containing PKM as a substrate. PKM is an unconventional feed ingredient that has high cellulose and mannan contents [5]. The low or high enzyme activity of the bacterial consortium indicates its ability to degrade cellulose and mannan in PKM.

The results, as shown in Figure 3, indicate a pattern in enzyme activities with different PKM substrate concentrations. Remarkably, the control group without the addition of PKM had the highest cellulase, mannanase, and protease activities. This suggests that the native conditions in the control favored enzyme activity to some extent. Among the PKM treatments, the addition of 20% PKM resulted in the highest cellulase activity compared with that of 15% and 25% PKM, implying an optimal substrate concentration for cellulase production or activation. On the other hand, 15% PKM treatment showed the highest mannanase and protease activities relative to 20% and 25% PKM, indicating that these enzymes perform better or are more efficiently produced under slightly lower PKM levels. The decrease in enzyme activities at 25% PKM could be due to substrate inhibition, nutrient imbalance, or physical effects such as substrate compaction limiting microbial growth or enzyme secretion.

These findings align with those of earlier studies where enzyme production typically peaks at an optimal substrate concentration and declines at higher levels due to such inhibitory effects [34]. The result of this study was higher than that of other studies that had cellulase activity of 0.204 U/mL [35], mannanase activity of 1.186 U/mL [36], and protease activity of 10.2 U/mL [37] from different bacteria using PKM as substrate. The differences in the results obtained can be caused by differences in the species of bacteria used and the percentage of PKM used as a substrate. Optimal conditions for the growth of both *L. fermentum* and *B. subtilis* and their enzyme secretion may have contributed to the superior results in this study compared to the previous study by Sheikhs *et al*. [38]. In addition, the use of freshly isolated or well-adapted strains could further enhance enzyme yields, as PKM adaptation as a substrate may increase polysaccharide and protein degradation efficiency [39].

The synergistic interaction between *L. fermentum* and *B. subtilis* likely played a crucial role in enhancing enzyme production. Bacterial consortia exhibit complementary metabolic activities, where one species may modify the substrate or produce metabolites that stimulate the growth or enzyme secretion of the other [40]. As observed in the present study, this mutualistic relationship can lead to higher overall enzyme yields compared to monocultures. The 1:1 ratio of *L. fermentum* to *B. subtilis* appears to optimize this synergy, particularly for cellulase production at higher PKM inclusion levels, while also supporting robust mannanase and protease activities at moderate PKM concentrations.

Furthermore, the physical and chemical characteristics of PKM may influence enzyme induction. PKM is rich in non-starch polysaccharides, such as cellulose and mannan, as well as proteins, which can induce microbial enzyme synthesis [41]. The differential enzyme activities observed at varying PKM concentrations suggest that substrate availability and accessibility may regulate the expression of specific enzymes. At 20% PKM, the higher cellulose content possibly provided sufficient induction for maximal cellulase production, whereas the substrate conditions were more favorable for mannanase and protease activity at 15% PKM.

### *In vitro* testing of bacterial combinations as probiotics

In this study, we evaluated not only the enzymatic activities of the isolates but also key probiotic traits, including bile salt tolerance, acid resistance, cell surface hydrophobicity, and pathogen inhibition. This integrated characterization strengthens our study’s originality and provides a foundation for practical application, as isolates exhibiting both strong enzymatic activity and robust probiotic traits are more likely to perform effectively when supplemented in poultry feed.

The test isolate was found to be negative for hemolysis, indicating that it does not lyse RBCs. This result suggests that the bacterium is nonpathogenic and safe for potential applications in food, feed, or probiotic formulations, as hemolytic activity is commonly associated with virulence in pathogenic bacteria. The absence of hemolytic activity aligns with the typical characteristics of LAB, including *Lactobacillus* species, which are generally regarded as safe and widely used as probiotics [42].

The testing of bacterial consortium as probiotics is carried out to determine their resistance *in vitro* in the livestock body. According to Srifani *et al*. [16], there are several criteria for microorganisms as probiotics, namely, having resistance to low pH (2.5), resistance to bile salts, having strong adhesive power (hydrophobicity) in the intestines, and being able to produce antibacterial activity against pathogens. In addition, they must not have any harmful side effects and be safe for the host.

Data, as shown in Table 3, show the ability of the bacterial consortium as a probiotic. The bacterial consortium was found to have 62.84% and 46.23% resistance to 0.3% and 0.5% bile salt concentration, respectively. The bile salt content of the small intestine typically varies between 0.03% and 0.3% [17]. The bacterial membrane may be harmed by bile acid; therefore, to remain viable in the intestine and fulfill their intended function, probiotic microorganisms must be resistant to bile salts [16]. Bacteria are considered resistant to bile salts if their survival rate is >50% at a specific bile salt concentration [43]. With a resistance percentage of over 50% at a bile salt concentration of 0.3%, the study’s findings indicated that the bacterial consortia in use had good resistance to bile salts. The ability to resist bile salts is primarily linked to bile salt hydrolase (BSH), an enzyme that defends cells from intracellular acidity induced by conjugated bile salts. BSH catalyzes the hydrolysis and deconjugation of glycine or taurine from the bile acid cholesterol backbone [16]. Probiotic bacteria should be resistant to low acid and high bile to some extent to benefit the health of the organism [44].

The low pH of gastric juice makes it effective at reducing microbial colonies. Therefore, probiotic microorganisms must have defense mechanisms to withstand the extreme pH swings that occur during digestion. In this study, the bacterial consortium was tested for its resistance at pH 2.5 according to the proventriculus and gizzard pH in chickens [45]. Based on the study, the bacterial consortium had a resistance of 70.60% at pH 2.5 after 3 h of incubation and 68.10% at an interval of 6 h after incubation. Mulaw *et al*. [46] stated that probiotic candidates have good resistance at low pH if they produce more than 50% resistance.

*L. fermentum* and *B. subtilis* exhibit tolerance to low pH due to a combination of active internal defense mechanisms and robust cell wall structures. Each strain possesses systems such as H^+^-ATPase proton pumps, arginine deaminase, and glutamate decarboxylase, which help maintain intracellular proton balance and prevent excessive acid accumulation. In addition, bacteria can protect themselves through active proton extrusion, proton consumption through decarboxylase reactions, and alkaline substance production, ensuring that their intracellular pH remains optimal under acidic conditions [16]. The Gram-positive cell walls of these bacteria contribute to acid resistance; their thick peptidoglycan layers and teichoic acids (TAs) provide mechanical strength and cell stability. TAs also support cation homeostasis, helping the cell withstand low pH stress [16]. Together, these physiological and structural features enable the *L. fermentum* and *B. subtilis* consortium to survive in acidic environments, such as the stomach or upper intestine, while retaining its probiotic functionality.

Probiotics work well because of their ability to adhere to intestinal epithelial cells. Hydrophobicity is an important characteristic that enhances the initial interaction between probiotic strains and host cells. This quality makes them the best choice to improve the health of their respective hosts and eliminate or reduce pathogen adhesion [47]. Bacterial adhesion and subsequent colonization in the intestine are more likely when there is a high percentage of hydrophobicity [48]. Based on the study results, the hydrophobicity value of the bacterial consortium was 83.75%. Nonpolar components, such as TA, lipoteichoic acid, lipopolysaccharides, and various surface proteins (S-layer), significantly contribute to the hydrophobic properties of the cell surface [49]. In addition, exopolysaccharide modulates bacterial attachment to host tissues. This result is higher than that of Wang *et al*. [50], who found that the hydrophobicity of the bacterial isolates LSG1-1 and LSG2-1, taken from the digestive tract of *Rhynchocypris lagowskii*, was 73.44% and 77.81%, respectively.

Both *L. fermentum* and *B. subtilis* exhibited high autoaggregation values, exceeding 70%. High autoaggregation indicates a strong ability of the bacterial cells to adhere to one another, which is considered an important trait for colonization and persistence in the gastrointestinal tract. This characteristic enhances the potential probiotic efficacy of the isolates, as bacteria with strong autoaggregation are more likely to form biofilms, compete with pathogens for adhesion sites, and maintain stability within the host environment [51]. The observed autoaggregation values suggest that both *L. fermentum* and *B. subtilis* possess favorable adhesive properties, supporting their suitability as probiotic candidates in food or feed applications.

The coaggregation between *L. fermentum* and *B. subtilis* was recorded at 78.13%, indicating a strong mutual interaction between the two strains. High coaggregation is considered a key probiotic feature because it promotes the establishment of mixed microbial communities and supports adherence to intestinal epithelial surfaces [52]. This trait may also help inhibit pathogenic microorganisms by occupying adhesion sites and forming a protective barrier within the gastrointestinal tract. The significant coaggregation observed indicates that these isolates could act synergistically, potentially enhancing their stability and functional performance when administered together as a probiotic.

The ability of probiotics to suppress the growth of harmful bacteria that can infect the digestive tract of poultry is one of the most crucial requirements of probiotics. When selecting probiotic strains, one factor that needs to be evaluated is their antimicrobial activity against infections [53]. Maintaining the balance of intestinal microflora and shielding the intestines against pathogens are two functions of the antimicrobial activity of probiotic microorganisms. The suppression of harmful bacteria (*S. aureus*, *S. enteritidis*, and *E. coli*) by the consortium bacterial isolates is depicted in Table 3. These three pathogenic bacteria are contagious and can lead to foodborne illnesses in humans after consumption, health issues, and even death in poultry. Based on the research results, the bacterial consortium tested had an inhibitory power against *E. coli* of 15.07 mm and an inhibitory power against *S. enteritidis* of 14.12 mm, and *S. aureus* of 17.12 mm. According to Winastri *et al*. [22], the ability of probiotic bacteria to suppress infections can be categorized as weak (≤5 mm), moderate (6–10 mm), strong (11–20 mm), or extremely strong (≥21 mm). Owing to their strong antibacterial activity, the evaluated consortium bacteria in this study may find application as probiotics. By adhering to the intestinal lining, probiotics can prevent pathogen colonization in the host intestine, either blocking pathogen interaction with specific host cell receptors or hindering their attachment through steric effects. In addition, probiotics produce antibacterial substances, such as bacteriocins, which enhance resistance to certain pathogens [54].

The resistance of consortium bacteria to heat was determined at 42°C, which is the average temperature of the chicken body and digestive tract [55]. The resistance of the bacterial consortium to 42°C, with a survival rate of 83.15%, indicates that the bacteria in the consortium can tolerate temperatures comparable to the physiological body temperature of chickens. This thermal resistance is important because probiotic candidates must survive and remain viable under host conditions to exert their beneficial effects. The high survival percentage suggests that the consortium is not only heat-tolerant but also potentially well-suited for colonization in the gastrointestinal tract of poultry.

### Enzyme activity of bacterial consortium in aqueous media

Microorganisms need specific environmental conditions, energy sources, and nutrients for growth and reproduction. Microbes adjust to the environment that best meets their short-term demands in the lab; the culture media must take these needs into account [56]. Microbes need the following nutrients to grow: carbon, nitrogen, metal elements such as Ca, Zn, Na, K, Cu, Mn, Mg, and Fe; non-metallic elements such as sulfur and phosphorus; vitamins, water, and energy [57].

One type of widely used media is liquid media. The propagation of bacteria with liquid media has the advantage of increasing the number of microbial cells faster than with solid media, and the propagation method is easier and more practical. So far, bacterial propagation has used commercial synthetic media, for example, using MRSB, where the price of commercial synthetic media is getting more expensive all the time. Therefore, to replace commercial synthetic media, an alternative use of organic materials that is cheaper and easier is needed.

Over the past few decades, a lot of research has investigated the use of different wastes as inexpensive media, which also has the added benefit of assisting in addressing the environmental issues brought on by the disposal of waste products [58]. The bacterial consortium was tested on several natural media to economically support its cultivation. The media tested were A0 (basic media MRSB), A1 (70% coconut water + 30% shrimp wastewater), A2 (70% tofu wastewater + 30% shrimp wastewater), and A3 (35% tofu wastewater + 35% coconut water + 30% shrimp wastewater).

As shown in Figure 4, it was found that the A1 treatment produced the highest enzyme activity, namely, cellulase, mannanase, and protease, of 18.39, 14.72, and 6.95 U/mL, respectively. These results indicate that media A1 (70% coconut water + 30% shrimp wastewater) can be used as a medium for bacterial cultivation. This can be because most of the elements required for bacterial development are present in coconut and shrimp water.

Coconut water is composed of glucose, fructose, and sucrose and serves as an energy source for bacteria, promoting growth [59]. Coconut water also contains essential amino acids, such as tyrosine, tryptophan, alanine, and valine, which are crucial for microbial growth and metabolism [60]. According to Dhanya *et al*. [61], coconut water has strong antioxidant properties; therefore, it may impact the dynamics of bacterial development. For example, the antioxidant qualities may boost beneficial strains of bacteria while suppressing harmful ones, thereby preserving a balanced microbial ecology.

Coconut water and shrimp water also have the potential to be used as natural media for bacterial growth. Shrimp water contains high levels of organic waste from uneaten feed and shrimp excretion, leading to increased levels of ammonia and nitrite [62]. This organic material provides essential nutrients for bacteria, facilitating their growth and metabolic activities.

The use of a natural medium composed of 70% coconut water and 30% shrimp wastewater offers a clear cost advantage over commercial MRSB. Shrimp wastewater is readily available as an industrial by-product at no cost, whereas coconut water is inexpensive and easy to obtain. In contrast, MRSB is relatively expensive, with 500 g costing approximately United States Dollar 120–200.

These findings highlight the economic feasibility of using coconut water and shrimp wastewater as a sustainable alternative medium for bacterial cultivation. The utilization of agro-industrial by-products such as shrimp wastewater and coconut water promotes waste valorization and supports more sustainable waste management practices. Furthermore, the abundant local availability of these resources highlights their promising potential for upscaling, making this natural medium a cost-effective and environmentally sustainable alternative for bacterial cultivation.

This study demonstrated that *L. fermentum* and *B. subtilis* can form a stable consortium that produces cellulase, mannanase, and protease with notable synergistic activity. The strains grew well in a natural medium consisting of 70% coconut water and 30% shrimp wastewater, highlighting their adaptability and potential for application in sustainable, low-cost substrates. Although the experiments were conducted under *in vitro* conditions without *in vivo* validation, and aspects such as scale-up feasibility and industrial application were beyond the current scope, the findings provide a strong foundation for future studies aimed at confirming probiotic functionality *in vivo* and optimizing production for practical applications.

## CONCLUSION

This study successfully demonstrated that a defined bacterial consortium comprising *L. fermentum* and *B. subtilis* exhibits superior enzymatic, probiotic, and growth characteristics compared to single-strain cultures. The consortium showed synergistic activity in producing cellulase (13.71 U/mL) and mannanase (17.05 U/mL), while *L. fermentum* alone yielded the highest protease activity (11.29 U/mL). When cultured in media containing PKM, the 1:1 ratio of *L. fermentum* and *B. subtilis* displayed optimal enzyme production at 15%–20% PKM, highlighting its potential to enhance nutrient digestibility in high-fiber poultry feeds. The bacterial combination also exhibited strong acid (70.6%) and bile (62.84%) tolerance, high hydrophobicity (83.75%), and substantial antibacterial activity against *E. coli*, *S. enteritidis*, and *S. aureus*, confirming its functional probiotic potential.

A key practical implication is the consortium’s ability to grow efficiently in low-cost natural media composed of 70% coconut water and 30% shrimp wastewater, producing high enzyme yields (cellulase 18.39 U/mL; mannanase 14.72 U/mL; protease 6.95 U/mL). This cost-effective substrate not only reduces dependence on commercial MRSB but also promotes sustainable waste utilization. The findings underscore the feasibility of developing an eco-friendly, economical probiotic-enzyme supplement for poultry nutrition and feed bioprocessing industries.

The strength of this study lies in its integration of enzymatic profiling, probiotic characterization, and media optimization, which together provide a comprehensive assessment of the consortium’s potential. However, the study was limited to *in vitro* conditions, and *in vivo* validation in poultry models is needed to confirm functional efficacy, gut colonization dynamics, and overall growth performance impacts.

Future research should explore scale-up fermentation kinetics, probiotic stability under pelleting conditions, and long-term effects on feed conversion efficiency, immunity, and microbiome modulation in poultry.

In conclusion, the *L. fermentum*–*B. subtilis* consortium represents a promising biotechnological innovation that synergizes enzyme production, probiotic function, and sustainable feed substrate utilization, offering a practical and environmentally responsible approach to improving poultry productivity and gut health.

## AUTHORS’ CONTRIBUTIONS

MM: Supervision and conceptualization. SA, HH, and GC: Conceptualization. ZZ: Data validation. GY: Methodology and formal analysis. AS: Data analysis and drafted and revised the manuscript. All authors have read and approved the final version of the manuscript.
